# Spatial transcriptomics reveals an SPP1-centered immune–fibrotic axis associated with fibrosis-related tissue remodeling in IgG4-related disease

**DOI:** 10.3389/fimmu.2026.1870169

**Published:** 2026-07-17

**Authors:** Motohisa Yamamoto, Ryuta Kamekura, Masaaki Uehara, Yuta Ichii, Kenichi Takano

**Affiliations:** 1 Department of Rheumatology, Allergy and Clinical Immunology, Institute of Medical Science, The University of Tokyo (IMSUT) Hospital, The Institute of Medical Science, The University of Tokyo, Tokyo, Japan; 2 Department of Otolaryngology-Head and Neck Surgery, Sapporo Medical University School of Medicine, Sapporo, Japan; 3 Division of Cancer Cell Biology, The Institute of Medical Science, The University of Tokyo, Tokyo, Japan

**Keywords:** fibrosis, IgG4-related disease, macrophages, spatial transcriptomics, T follicular helper cells

## Abstract

IgG4-related disease (IgG4-RD) is a fibroinflammatory condition characterized by progressive organ damage; however, the spatial organization linking immune dysregulation to fibrotic remodeling remains incompletely understood. In this study, we performed spatial transcriptomic analysis of submandibular gland tissues from patients with IgG4-RD representing two distinct fibrosis-associated tissue states. Gene expression data were normalized and z-scored, and immune- and fibrosis-related module scores were calculated. Fibrotic niches were defined based on COL1A1 expression and fibroblast signatures, and spatial co-localization and correlation analyses were conducted. Fibrosis-high tissue regions exhibited enrichment of SPP1-expressing macrophages that co-localized with COL1A1 and fibroblast-rich areas, consistent with a spatially defined fibrotic niche. These regions were associated with increased expression of PDGFB and TGF-β–related molecules, which correlated with fibrosis-related signatures. Immune network analysis suggested remodeling of the immune microenvironment, with expansion and integration of Tph-associated populations. A preTfh-to-Tph axis became more apparent in fibrosis-high lesions, accompanied by reduced germinal center B-cell signatures and enhanced extrafollicular activation. Exhaustion-associated programs, including PDCD1 and TOX, were associated with T-cell differentiation states and SPP1-related signatures. Together, these findings identify an SPP1-associated immune–fibrotic niche and suggest a spatially coordinated relationship between immune remodeling and fibrosis in IgG4-RD. This hypothesis-generating framework provides insights into fibrosis-associated tissue remodeling and may inform future strategies for disease stratification and therapeutic targeting.

## Graphical abstract

**Figure:**
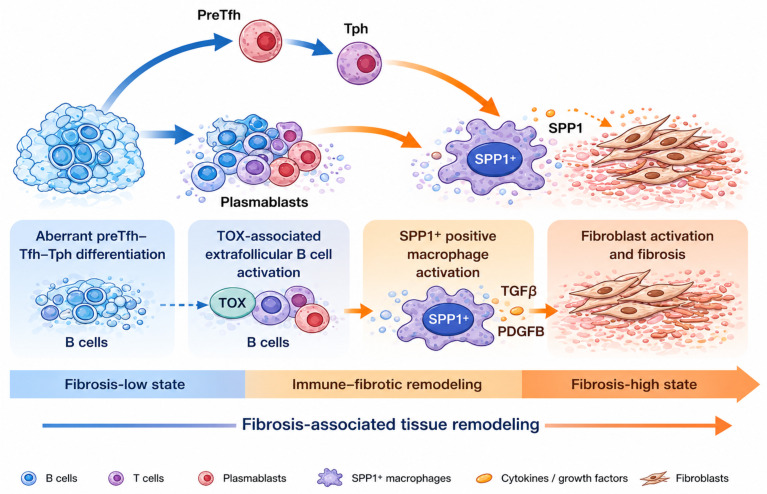

## 1 Introduction

IgG4-related disease (IgG4-RD) is a chronic systemic inflammatory disorder characterized by multiorgan enlargement, lymphocytic and plasma cell infiltration, the formation of enlarged tertiary lymphoid structures, and characteristic fibrotic changes such as storiform fibrosis (1, 2). While the disease responds well to glucocorticoids, it is frequently recurrent and requires long-term management (3). In particular, fibrosis associated with disease progression is a major cause of irreversible organ damage, and elucidating its molecular mechanisms remains a critical challenge.

Fibrosis in IgG4-RD has traditionally been regarded as a downstream consequence of persistent inflammation. However, emerging evidence suggests that fibrosis is not merely an end-stage outcome, but rather represents a distinct microenvironment formed through local cell–cell interactions, with the potential to actively modulate immune responses (4, 5). Nevertheless, the spatial and cellular framework within which fibrosis develops, and how it interacts with the immune microenvironment, remain poorly understood.

Recent studies across multiple fibrotic diseases have identified SPP1-positive macrophages as key drivers of fibroblast activation and extracellular matrix remodeling (6–9). However, whether SPP1-associated fibrotic niches exist in IgG4-RD and how they interact with local immune architecture remain unknown.

Meanwhile, abnormalities in T cell subsets, particularly follicular helper T (Tfh) cells (10) and peripheral helper T (Tph) cells (11), have been identified as key immunological features of this disease. In addition, dysregulation of germinal center responses and reprogramming of T cell differentiation have been suggested; however, how these immunological alterations relate to tissue architecture, especially fibrosis, remains unclear.

To address these gaps, we performed spatial transcriptomic analysis of IgG4-RD lesions to delineate the coordinated relationship between immune cell dynamics and fibrotic remodeling. By integrating spatial gene expression patterns with immune and stromal features, we sought to define the structural and functional organization of the disease microenvironment and to establish a spatially resolved framework linking immune dysregulation to fibrosis.

## 2 Methods

### 2.1 Subjects and tissue samples

This study included submandibular gland tissues from two patients with IgG4-related disease (IgG4-RD) collected for diagnostic purposes at Sapporo Medical University between October and November 2023. Both patients fulfilled the 2020 revised comprehensive diagnostic criteria for IgG4-RD (12). The two lesions were analyzed as representative fibrosis-low and fibrosis-high cases to explore spatial changes associated with disease progression.

The spatial transcriptomic datasets analyzed in the present study were generated from the same specimens included in our previously published spatial transcriptomic studies of IgG4-RD (13, 14). However, the present study addressed a distinct biological question focused on fibrosis-associated spatial microenvironments and immune–fibrotic remodeling. Whereas our previous studies primarily investigated T-cell organization, germinal center dynamics, and Tph-associated immune responses, the current study focused on SPP1-centered fibrotic niches, immune–stromal interactions, and spatial mechanisms underlying disease progression. All analyses presented in this manuscript were newly performed for these objectives.

Importantly, both patients were treatment-naïve at the time of biopsy, and tissue samples were obtained before the initiation of systemic glucocorticoid or immunosuppressive therapy (**Table 1**).

**Table 1:** Clinical characteristics of the two patients with IgG4-related disease.

Characteristic	Case 1 (fibrosis-low)	Case 2 (fibrosis-high)
Age (years)	58	68
Sex	M	M
Disease duration (months)	6	5
Serum IgG4 (mg/dL)	682	1123
Organ involvement	Lacrimal gland	Lacrimal gland
	Submandibular gland	Submandibular gland
	Parotid gland	Parotid gland
	Bronchus	Sublingual gland
		Thyroid gland
		Aorta
Treatment before biopsy	None	None

### 2.2 Spatial transcriptomics analysis

Spatial transcriptomic analysis was performed using the 10x Genomics Visium Spatial Gene Expression for FFPE platform (10x Genomics, Pleasanton, CA, USA) (13). Libraries were prepared according to the manufacturer’s protocol and sequenced using the NovaSeq 6000 system (Illumina, San Diego, CA, USA) (14). Spatially barcoded gene expression matrices were generated and used for downstream spot-level analyses.

### 2.3 Data preprocessing and normalization

Spatial gene expression data were analyzed in R (version 4.5.2) using the Seurat package (version 5.4.0). Gene expression counts were normalized for library size and log-transformed. For module-based analyses, gene-wise z-scores were calculated across all spots within each sample. These normalized and standardized values were used for spatial visualization, module scoring, correlation analyses, and definition of high-expression regions.

### 2.4 Definition of immune cell and fibrosis-related modules

Immune and stromal cell programs were defined using curated gene signatures based on previous reports and biological relevance (15–18). These included T cell subsets, B cell and plasma cell programs, macrophage-related signatures, fibroblast-related signatures, fibrosis-associated macrophages, and exhaustion-related programs (**Supplementary Table 1**). Module scores were calculated as the mean z-score of genes included in each module. Scores were calculated only when at least two genes from a given module were detected in the dataset. The fibrosis-associated macrophage (FAM) module was defined using CD163, MRC1, CCL18, and TGFB1, based on previous studies identifying profibrotic macrophage populations involved in tissue remodeling and fibrosis (19, 20). Notably, SPP1 was not included in the predefined FAM module and was subsequently evaluated independently because of its reported role as a key profibrotic macrophage marker across multiple fibrotic diseases (21, 22).

### 2.5 Assessment of fibrosis and classification of fibrosis-related lesions

Fibrosis was evaluated using COL1A1 expression and fibroblast module scores. Based on these fibrosis-related features, the two cases were classified as fibrosis-low and fibrosis-high lesions. Fibrosis-high spots were defined as spots within the top 20% of the fibrosis score and were used to identify spatial fibrotic niches.

### 2.6 Spatial niche and co-localization analyses

SPP1 expression was analyzed as an independent profibrotic marker using both log-normalized expression values and z-scores. High-SPP1, high-COL1A1, and fibroblast-rich regions were defined using upper-percentile thresholds. Spatial overlap among these regions was assessed to evaluate the formation of an SPP1-associated fibrotic niche. Distances from high-scoring immune or stromal module spots to fibrosis-high spots were calculated using nearest-neighbor analysis.

### 2.7 Correlation analyses

Associations between fibrosis scores, SPP1 expression, profibrotic mediators, and immune or stromal module scores were evaluated using correlation analyses. Spearman’s rank correlation was used for associations involving fibrosis scores and spatial module scores. Correlation coefficients and corresponding p values were calculated at the spot level within each case.

### 2.8 Definition of follicular and extrafollicular immune programs

Follicular immune activity was assessed using Tfh and germinal center B cell module scores. Extrafollicular immune activity was evaluated using Tph, activated B cell, extrafollicular B cell, and plasmablast-related module scores. A preTfh-to-Tph index was calculated from standardized preTfh and Tph module scores to evaluate spatial remodeling of T cell differentiation states and their relationship with B cell programs.

### 2.9 Inference of cell–cell communication from spatial transcriptomic data

To explore potential intercellular communication within the spatial tissue microenvironment, ligand–receptor interaction analysis was performed using CellChat (23). Because the analysis was based on spot-level spatial transcriptomic data rather than single-cell RNA sequencing, CellChat was used as an exploratory framework to infer communication between spatially defined module-high spot populations.

For each case, spots were assigned to representative cell-state groups according to high module scores, including fibrosis-associated macrophages, M2-like macrophages, fibroblasts, Tph cells, germinal center B cells, and plasmablasts. Spots not assigned to these groups were excluded from the CellChat analysis. A sparse expression matrix restricted to CellChat ligand–receptor genes was generated from the 10x Genomics matrix file to reduce memory usage. Expression values were library-size normalized and log-transformed before analysis.

CellChat objects were created using the normalized spot-level expression matrix and the corresponding module-defined group annotation. Human ligand–receptor interactions from the CellChat database were used. Communication probabilities were computed using normalized expression values, and interactions were filtered by a minimum group size threshold. Inferred interactions were summarized at the pathway level, and total interaction number and communication strength were visualized as network plots. Particular attention was paid to SPP1-, TGFB-, PDGF-, CXCL13-, and IL21-related ligand–receptor interactions and their potential roles in linking macrophage-rich, fibroblast-rich, and lymphoid immune regions.

### 2.10 Statistical analysis

Comparisons between spatially defined groups were performed using non-parametric tests, including the Wilcoxon rank-sum test where appropriate. Correlation analyses were performed using Spearman’s or Pearson’s correlation coefficients as specified above. P values were two-sided, and p < 0.05 was considered statistically significant. All analyses were performed using R.

## 3 Results

### 3.1 Staging based on fibrosis and spatial heterogeneity

**Figure 1:**
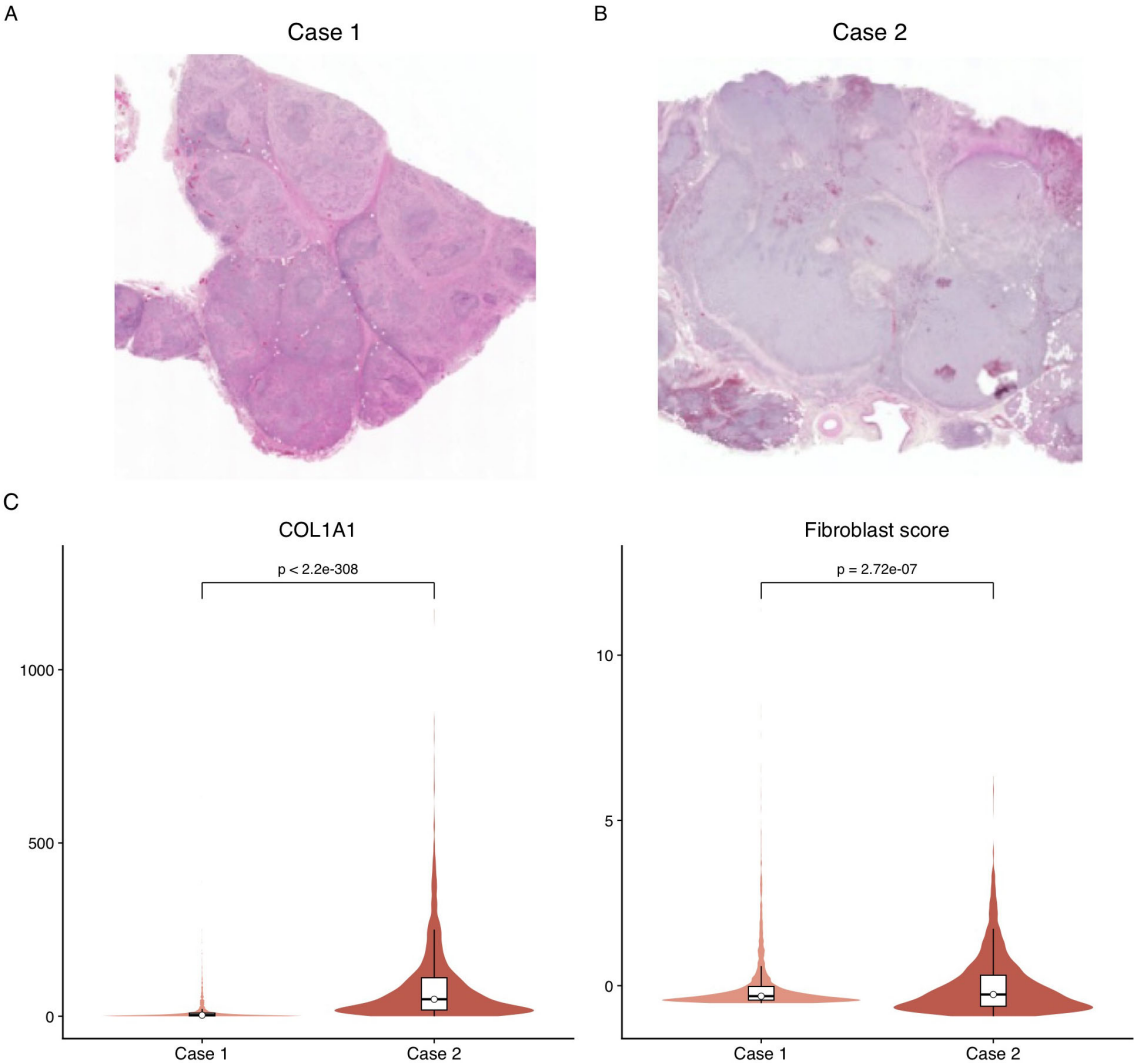
Fibrosis-based classification of two submandibular gland lesions. **(A)** Hematoxylin and eosin-stained sections from Case 1 and Case 2. **(B)** Quantitative comparison of COL1A1 expression and fibroblast module scores between the two lesions. The fibroblast score was calculated using a fixed fibrosis-associated gene module (COL1A1, COL3A1, FN1, POSTN, FAP, BGN, and TIMP1). P values were calculated using the Wilcoxon rank-sum test on the original spot-level data. For visualization, the upper y-axis range in each panel was adjusted using the 99.5th percentile. Based on fibrosis-related gene expression, Case 1 and Case 2 were defined as fibrosis-low and fibrosis-high lesions, respectively.

A comparison of fibrosis-related gene expression revealed that both COL1A1 expression and fibroblast module scores were higher in Case 2 than in Case 1. Accordingly, Case 1 was defined as a fibrosis-low lesion, whereas Case 2 was defined as a fibrosis-high lesion (**Figure 1**).

To validate the fibrosis classification based on transcriptomic features, Masson Trichrome staining was performed on the corresponding tissue sections. Consistent with the spatial transcriptomic findings, the fibrosis-high lesion showed markedly increased collagen deposition compared with the fibrosis-low lesion (**Supplementary Figure 1**), supporting the classification based on COL1A1 expression and fibroblast module scores.

Spatial analysis demonstrated that fibrosis-related signals were relatively dispersed in Case 1, whereas in Case 2 they were concentrated within restricted regions, suggesting differences in the spatial organization of fibrosis-related tissue microenvironments between fibrosis-low and fibrosis-high lesions.

### 3.2 Formation of an SPP1-centered fibrotic niche

**Figure 2:**
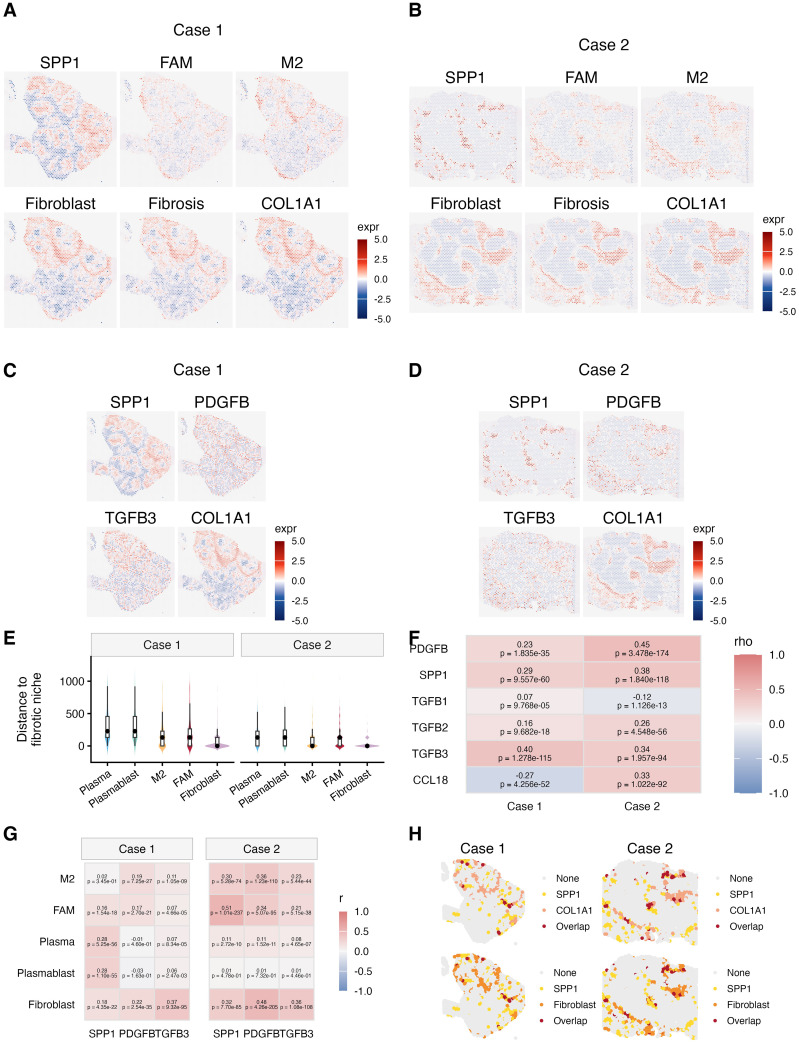
Spatial organization of a fibrosis-associated niche in IgG4-related disease. **(A, B)** Spatial distribution of SPP1, fibrosis-associated macrophages (FAM), M2 macrophages, fibroblasts, fibrosis module scores, and COL1A1 in Case 1 and Case 2. Values are shown as z-scores. **(C, D)** Spatial expression of SPP1, PDGFB, TGFB3, and COL1A1, illustrating the relationship between profibrotic mediators and fibrotic regions. **(E)** Distance analysis of high-scoring cell populations from fibrotic niches. Distances were calculated from feature-high spots to the nearest fibrosis-high spot. **(F)** Correlation between fibrosis scores and expression of PDGFB, SPP1, TGFB1, TGFB2, TGFB3, and CCL18. Correlation coefficients and exact p values are shown. **(G)** Correlation between profibrotic gene expression (SPP1, PDGFB, TGFB3) and cell-type/module scores (M2, FAM, plasma cells, plasmablasts, fibroblasts). **(H)** Spatial overlap analysis of SPP1 with COL1A1 or fibroblast-rich regions, highlighting their spatial co-localization. These results demonstrate that SPP1-positive macrophage-rich regions spatially coincide with fibrotic niches, supporting the presence of a localized fibrosis-associated microenvironment in IgG4-RD.

We next examined the spatial organization of profibrotic molecules and cell populations. In Case 1, SPP1 and COL1A1 showed limited spatial overlap. In contrast, in Case 2, regions of high SPP1 expression strongly co-localized with COL1A1 and fibroblast-rich areas, forming a unified spatial structure (**Figures 2A, B**).

Furthermore, SPP1 expression was closely associated with fibrosis-associated macrophages (FAM) and M2-like macrophages in Case 2, whereas such convergence was less evident in Case 1. PDGFB and TGFB3 were also enriched in SPP1-high regions (**Figures 2C, D**). These observations were supported by correlation analyses showing significant associations between fibrosis scores and SPP1, PDGFB, and TGF-β–related molecules (**Supplementary Figure 2**).

### 3.3 Cellular composition and spatial distribution of the fibrotic niche

Distance analysis revealed that in Case 2, FAM, M2 macrophages, and fibroblasts were preferentially localized near fibrotic niches, whereas plasmablasts and plasma cells were distributed more distantly (**Figure 2E**).

In contrast, Case 1 exhibited a more dispersed distribution of these cell populations, without clear spatial enrichment. These fibrosis-associated cell populations were preferentially enriched in extrafollicular regions, particularly in Case 2 (**Supplementary Figure 3**).

### 3.4 Molecular interactions underlying fibrotic niche formation

Correlation analyses demonstrated that SPP1 was associated with macrophage-related modules, whereas PDGFB showed stronger associations with fibroblast-related signatures (**Figures 2F, G**).

Spatial overlap analysis further revealed that SPP1-high regions coincided with COL1A1 and fibroblast-rich areas, particularly in Case 2 (**Figure 2H**). In addition, SPP1 receptor components, including CD44 and integrin αvβ, showed similar spatial distributions (**Supplementary Figure 4**), suggesting the presence of a functional SPP1 signaling axis within the fibrotic niche.

### 3.5 Reorganization of the immune cell network

**Figure 3:**
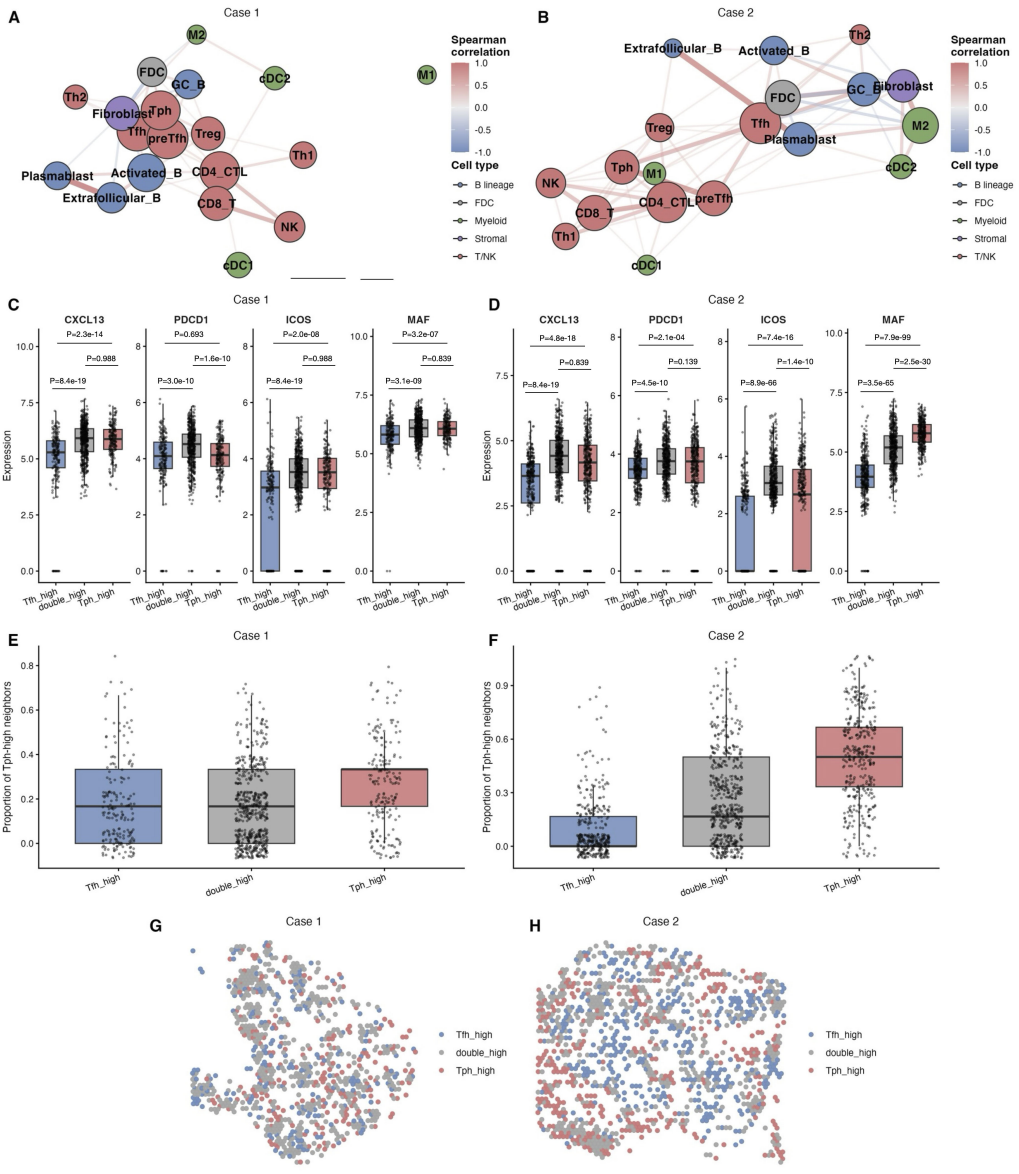
Progressive reorganization of immune cell networks and T cell differentiation axis in IgG4-related disease. **(A, B)** Spearman correlation–based immune cell interaction networks in Case 1 (fibrosis-low) and Case 2 (fibrosis-high). Node size reflects module activity, and edge thickness represents correlation strength. In Case 1, Tfh, preTfh, and Tph form a loosely connected cluster without clear directionality. In contrast, Case 2 shows a reorganized network in which Tph-associated populations expand and become more broadly integrated into the immune network, indicating remodeling of the immune architecture during fibrosis-associated tissue remodeling. **(C, D)** Expression of T cell differentiation–related genes (CXCL13, PDCD1, ICOS, and MAF) across Tfh_high, double_high, and Tph_high states in Case 1 **(C)** and Case 2 **(D)**. Case 2 demonstrates a clearer stratification consistent with a preTfh-to-Tph differentiation axis. **(E, F)** Proportion of Tph-high neighboring spots across the three states. In Case 2, Tph_high regions show significantly increased spatial clustering compared with Case 1, indicating spatial consolidation of Tph-associated microenvironments during progression. **(G, H)** Spatial distribution of Tfh_high, double_high, and Tph_high states in Case 1 **(G)** and Case 2 **(H)**. While Case 1 exhibits a dispersed pattern, Case 2 shows spatial segregation and clustering, consistent with structured immune reorganization. Collectively, these findings indicate that disease progression is associated with coordinated remodeling of immune networks, spatial consolidation of Tph niches, and strengthening of the preTfh-to-Tph differentiation axis.

Correlation-based network analysis revealed distinct immune architectures between the two cases. In Case 1, GC B cells formed a central hub, connected to Tfh-, preTfh-, and Tph-related programs, suggesting a germinal center–associated immune structure (**Figure 3A**).

In contrast, Case 2 exhibited a reorganized network in which GC B cell centrality was reduced and interactions were redistributed toward macrophage- and fibroblast-associated components (**Figure 3B**).

These network-level changes were supported by spatial distribution patterns of T cell subsets. In Case 2, preTfh-, Tfh-, and Tph-related programs showed localized clustering despite an overall reduction in signal intensity, whereas Case 1 exhibited a more diffuse distribution (**Supplementary Figure 5**).

### 3.6 Strengthening of the preTfh to Tph differentiation axis

To evaluate the presence of a differentiation axis from preTfh to Tph, we performed an analysis based on module scores. In Case 1, the relationships among preTfh, Tfh, and Tph were weak, and no clear directional axis was observed. In contrast, in Case 2, these populations exhibited a structured distribution pattern, indicating spatial and functional reorganization. Expression of T-cell differentiation–related genes, including CXCL13, PDCD1, ICOS, and MAF, showed clearer stratification across Tfh_high, double_high, and Tph_high states in Case 2 than in Case 1 (**Figures 3C, D**). In addition, Tph_high regions exhibited significantly increased neighboring Tph_high spots in Case 2, indicating enhanced spatial clustering of Tph-associated microenvironments (**Figures 3E, F**). Spatial mapping further demonstrated segregation and consolidation of Tfh_high, double_high, and Tph_high states in Case 2, whereas these states remained more diffusely distributed in Case 1 (**Figures 3G, H**).

Consistent with these findings, correlation-based network analysis further supported a coordinated alignment of preTfh-, Tfh-, and Tph-related programs in Case 2 (as shown in **Figure 3**), suggesting the emergence of a structured differentiation axis. These findings indicate that Tph-associated populations are more extensively integrated into the reorganized immune microenvironment in fibrosis-high lesions.

As the preTfh-to-Tph index increased, Case 2 demonstrated a decrease in germinal center B-cell signatures and a concomitant increase in extrafollicular B-cell and plasmablast-related programs (**Supplementary Figure 6**). These changes were continuous and consistent with a progressive shift in B-cell differentiation states.

**Figure 4:**
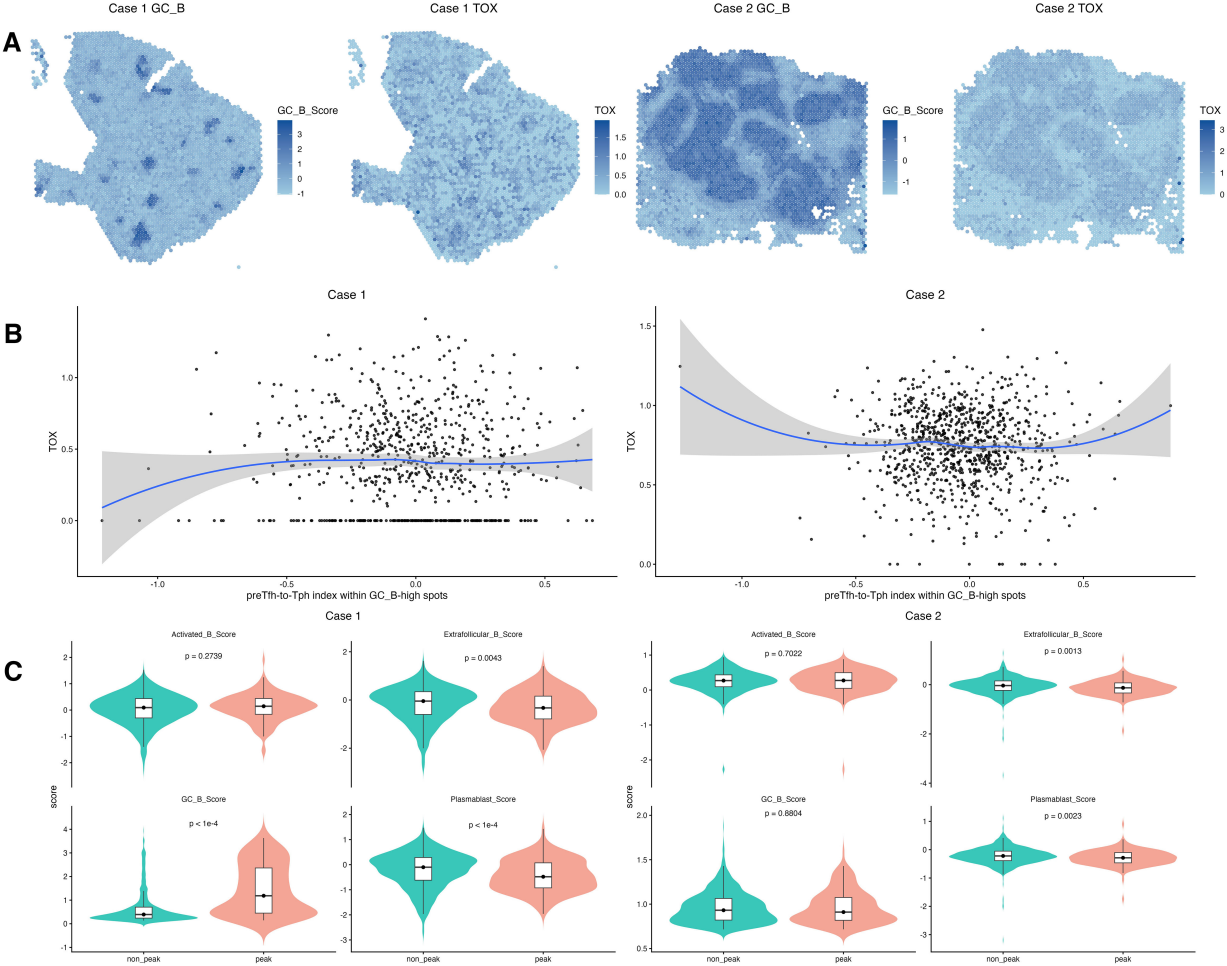
GC-like niche formation and TOX-associated immune remodeling in IgG4-RD. **(A)** Spatial distribution of GC_B_Score and TOX expression in submandibular gland lesions from an early-stage case (Case 1) and an advanced-stage case (Case 2). In the early case, GC_B and TOX signals show partially overlapping spatial patterns, whereas in the advanced case these patterns become more diffuse and partially dissociated, indicating reorganization of exhaustion-related programs during disease progression. **(B)** Relationship between TOX expression and the preTfh-to-Tph index within GC_B-high regions. In the advanced case, TOX exhibits a non-linear association with the preTfh–Tph axis, whereas this relationship is less apparent in the early case, suggesting stage-dependent organization of T-cell states. **(C)** Comparison of B-cell program scores between high and low preTfh-to-Tph index regions. In the early case, peak regions show increased GC_B scores and reduced plasmablast and extrafollicular B-cell signatures, whereas these differences are attenuated in the advanced case, indicating stage-dependent remodeling of B-cell programs along the preTfh-to-Tph axis.

To further characterize the relationship between the preTfh-to-Tph axis and exhaustion-related programs, we integrated spatial distribution, correlation analysis, and functional comparisons. Spatial mapping revealed partially overlapping but distinct patterns of GC_B and TOX expression (**Figure 4A**). TOX expression showed modest associations with the preTfh-to-Tph index, with case-dependent differences (**Figure 4B**).

Furthermore, regions with high preTfh-to-Tph index (“peak” regions) exhibited reduced germinal center B-cell signatures and increased plasmablast-related programs, consistent with a functional shift toward extrafollicular responses (**Figure 4C**).

### 3.7 Exhaustion-related programs and immune remodeling

In Case 1, TOX and PDCD1 were strongly associated with GC_B and preTfh-related programs, whereas in Case 2, these associations were reorganized, with PDCD1 remaining strongly associated with preTfh and Tph-related programs while TOX associations were markedly attenuated. In contrast, NR4A1 exhibited relatively weak and broadly distributed associations across both cases.

These findings suggest that exhaustion-related programs are not simply amplified in fibrosis-high lesions, but are instead dynamically reconfigured in association with immune microenvironmental remodeling (**Supplementary Figure 7**).

### 3.8 Integrated model of immune–fibrotic remodeling

Integrating the findings from **Figures 2**-**4**, fibrosis-low lesions exhibited relatively dispersed immune and fibrosis-related components organized around a germinal center–associated immune architecture. In contrast, fibrosis-high lesions showed a spatially defined fibrotic niche centered on SPP1, characterized by convergence of macrophages, fibroblasts, and extracellular matrix-associated programs.

These differences were accompanied by a shift from GC_B- and Th2-associated interactions to macrophage-centered communication networks (**Supplementary Figure 8**), along with strengthening of the preTfh-to-Tph differentiation axis, attenuation of germinal center B-cell responses, and enhancement of extrafollicular immune programs.

## 4 Discussion

In this study, we conducted an integrated analysis of the relationship between fibrosis and immune abnormalities in IgG4-related disease (IgG4-RD) using spatial transcriptomics, thereby elucidating patterns of microenvironmental reorganization associated with fibrosis-related tissue remodeling.

First, a key finding of this study is that fibrosis does not simply progress diffusely as a result of inflammation, but rather is established as a spatially organized “fibrotic niche.” In particular, we identified a niche characterized by the convergence of SPP1-positive macrophages, fibroblasts, and extracellular matrix-associated molecules, including COL1A1, accompanied by enrichment of PDGF- and TGF-β–related signaling.

Recent studies have highlighted SPP1^+^ macrophages as key drivers of fibrosis across multiple tissues (24, 25), promoting fibroblast activation and extracellular matrix deposition (6, 7). In particular, SPP1^+^ macrophages have been shown to induce fibroblast-to-myofibroblast transformation under hypoxic conditions (8), and their widespread involvement across tissues and disease contexts has been increasingly recognized (9).

Our findings extend these observations by demonstrating that such macrophage-driven programs are not only present but are spatially organized into discrete niches within IgG4-RD lesions. This spatial organization suggests that fibrosis is actively maintained through localized cellular interactions rather than passive accumulation.

Importantly, our results go beyond spatial co-localization. Ligand–receptor analysis suggested that macrophage-associated populations, particularly M2-like and fibrosis-associated macrophages, act as central hubs of intercellular communication in fibrosis-high lesions. These interactions were enriched for SPP1-, PDGF-, and TGF-β–related signaling pathways, supporting a model in which macrophage-derived signals orchestrate fibroblast activation and niche maintenance. Although these analyses are inferential, they provide a systems-level framework linking molecular signaling to spatial organization.

Next, we demonstrate that fibrosis and immune architecture are tightly interconnected. In fibrosis-high lesions, the immune cell network was restructured, with Tph-associated populations expanding and becoming more broadly integrated into the immune network. This change reflects a shift from a germinal center–associated immune structure toward a more distributed, tissue-based immune program.

Tph cells, which lack CXCR5 but retain B-cell helper function, have been implicated in extrafollicular immune responses in autoimmune diseases (26). Previous studies have suggested that spatial organization of Tfh and Tph populations regulates these responses (27–29). Our findings extend this concept by showing that such reorganization occurs in parallel with fibrotic niche formation, suggesting a coordinated remodeling of immune and stromal compartments.

A particularly notable finding is the emergence of a differentiation axis from preTfh to Tph in fibrosis-high lesions. This transition was associated with a reduction in germinal center B-cell signatures and a concomitant increase in extrafollicular B-cell and plasmablast programs, consistent with prior observations in autoimmune conditions (30–32), including dysregulation of germinal center responses (33). Furthermore, ligand–receptor analysis indicated that Tph-associated populations are integrated into macrophage- and fibroblast-centered communication networks, linking T-cell differentiation to fibrotic niche formation.

Importantly, this differentiation axis was closely associated with exhaustion-related transcriptional programs. In particular, PDCD1, TOX, and NR4A1 showed associations with Tph-related programs. Recent studies have established TOX and NR4A family transcription factors as key regulators of T-cell exhaustion and differentiation states (34–36), including transcriptional network regulation and stemness-associated exhaustion programs (37, 38). Our findings extend this framework by suggesting that these transcriptional programs may function as dynamic regulators linking T-cell differentiation with macrophage-driven fibrotic remodeling in IgG4-RD.

Our findings are broadly consistent with previous single-cell transcriptomic studies of IgG4-RD salivary gland tissues, which demonstrated expansion of helper T-cell populations, activated macrophages, and aberrant B-cell differentiation programs. These studies highlighted the cellular heterogeneity of immune populations involved in IgG4-RD pathogenesis. The present study extends these observations by providing spatial context, demonstrating how these immune populations are organized within tissue microenvironments and how they interact with fibrosis-associated niches. In particular, the identification of an SPP1-centered immune–fibrotic niche suggests a spatial framework linking macrophage activation, immune remodeling, and fibrosis that cannot be readily resolved by dissociative single-cell approaches alone. Whereas previous single-cell studies primarily characterized cellular composition and transcriptional states, the present study provides a complementary spatial framework by identifying immune–fibrotic niches within intact tissue architecture.

Integrating these findings, we propose that fibrosis-high lesions in IgG4-RD are characterized by a coordinated shift from a germinal center–oriented immune architecture toward a macrophage-centered fibrotic microenvironment. This tissue state is characterized by spatial convergence of SPP1^+^ macrophages and fibroblasts, reorganization of immune cell networks, and remodeling of T-cell and B-cell differentiation programs. Together, these results support the concept of an “immune–fibrotic link,” in which immune dysregulation and fibrosis are spatially and functionally coupled processes that mutually reinforce each other (39–41). Importantly, the fibrosis-low and fibrosis-high designations used in this study reflect relative differences in fibrosis-related tissue features rather than definitive chronological stages of disease progression. Although symptom duration was similar between the two patients, the observed differences cannot be directly attributed to disease duration alone. Therefore, the proposed model should be interpreted as a framework describing distinct fibrosis-associated tissue states that require validation in larger cohorts.

This study has several limitations. First, the analysis is based on a limited number of cases, and validation in larger cohorts will be necessary to establish the generalizability of these findings.

Second, as this is a cross-sectional study, causal relationships between immune remodeling and fibrosis cannot be definitively established. Longitudinal and functional studies will be required to clarify temporal dynamics and mechanistic pathways.

Third, the ligand–receptor analysis performed in this study is based on spatial transcriptomic data and therefore represents an inference of potential intercellular communication rather than direct measurement. In particular, because spatial transcriptomic spots contain multiple cell types, the analysis should be interpreted as a pseudo-cellular approximation of cell–cell interactions. Future studies using single-cell approaches or functional assays will be necessary to validate these predicted interactions.

Fourth, the current spatial transcriptomic dataset did not permit robust analysis of IGHG4 transcript distribution. Therefore, we were unable to directly evaluate the spatial relationship between IgG4-producing plasma cells and fibrosis-associated niches.

Fifth, direct protein-level validation of SPP1-positive macrophages was not feasible because no additional tissue material remained after diagnostic evaluation and spatial transcriptomic analysis. Therefore, the proposed spatial co-localization of SPP1-positive macrophages and fibrotic niches should be interpreted as a transcriptomics-based observation that warrants future validation using immunohistochemistry or multiplex immunofluorescence.

Finally, although the present study provides a comprehensive spatial and systems-level framework, direct functional validation using patient-derived cells or *in vivo* models was not performed. Therefore, the proposed immune–stromal interactions and their role in fibrosis should be considered as testable hypotheses that warrant further experimental investigation.

## 5 Conclusion

In this study, we conducted an integrated analysis of the relationship between fibrosis and immune abnormalities in IgG4-related diseases using spatial transcriptomics, thereby elucidating patterns of microenvironmental reorganization associated with fibrosis-related tissue remodeling. In fibrosis-high lesions, a fibrotic niche centered on SPP1 was formed, and a structure was observed in which molecular, cellular, and immune networks spatially converged. Concurrently, the establishment of a differentiation axis from preTfh to Tph, the activation of exhaustion-related programs, and the reorganization from germinal center B-cell responses to extrafollicular responses proceeded in a coordinated manner. In summary, fibrosis-associated tissue remodeling in IgG4-RD appears to involve coordinated interactions among fibrotic niche formation, immune-cell differentiation, and immune network reorganization. This study presents a new conceptual framework that comprehensively addresses fibrosis and immune abnormalities, while also providing a foundation for the identification of future therapeutic targets.

## Data Availability

The data underlying this article will be shared on reasonable request to the corresponding author.

## Supplementary material

Supplementary Figure 1 Histopathological validation of fibrosis classification by Masson Trichrome staining. Representative Masson Trichrome staining images of the fibrosis-low lesion (Case 1) and fibrosis-high lesion (Case 2). The fibrosis-high lesion showed substantially greater collagen deposition than the fibrosis-low lesion, consistent with the classification based on COL1A1 expression and fibroblast module scores derived from spatial transcriptomic analysis.

Supplementary Figure 2 Correlation between fibrosis-associated cells and molecular signatures. Heatmap showing Spearman correlation coefficients (rho) between fibrosis-related cellular modules (macrophage, M2, FAM, fibroblast) and plasma cell–related populations in Case 1 and Case 2. Fibroblast and macrophage-associated modules show progressively stronger positive correlations in Case 2, consistent with enhanced coupling between immune and fibrotic programs during disease progression.

Supplementary Figure 3 Preferential enrichment of fibrosis-associated cells in extrafollicular regions. Violin plots comparing module scores of fibrosis-associated cell populations (FAM, fibroblast, M2, macrophage, plasma cells, and plasmablasts) between intrafollicular and extrafollicular regions in Case 1 and Case 2. Across both cases, but more prominently in Case 2, these cell populations are enriched in extrafollicular regions, supporting the association between fibrosis and extrafollicular immune responses.

Supplementary Figure 4 Spatial expression of SPP1 receptor components. Spatial expression maps of CD44 and integrin αvβ receptors (αvβ1 and αvβ5) in Case 1 and Case 2. These receptors display overlapping spatial patterns with fibrosis-associated regions, supporting the presence of a functional SPP1 signaling axis within the fibrotic niche.

Supplementary Figure 5 Spatial organization of Tfh-related programs. Spatial distribution of preTfh, Tfh, and Tph module scores. In the early case, these programs are broadly distributed, consistent with active immune remodeling, whereas in the advanced case their overall intensity is reduced, suggesting diminished canonical Tfh organization despite strong GC-like features.

Supplementary Figure 6 B-cell program dynamics along the preTfh–Tph axis. B-cell–related module scores along the preTfh-to-Tph index. In Case 2, increasing preTfh-to-Tph index was associated with reduced GC_B signatures and relative enhancement of extrafollicular B-cell and plasmablast-related programs, supporting a shift in B-cell differentiation states.

Supplementary Figure 7 Association between exhaustion-related genes and immune modules. Spearman correlation heatmaps between exhaustion-related genes (including TOX, PDCD1, TIGIT, NR4A1) and immune module scores. In the early case, TOX and related exhaustion markers are positively associated with preTfh/Tph programs and B-cell modules, whereas these associations shift toward Tph-related programs in the advanced case, suggesting dynamic reconfiguration of exhaustion-related programs during immune microenvironmental remodeling.

Supplementary Figure 8 CellChat-inferred communication networks in IgG4-RD. **(A, B)** Inferred interaction networks in Case 1 **(A)** and Case 2 **(B)**. Nodes represent cell-state groups, and edge thickness indicates inferred communication probability. Case 1 shows a network characterized by prominent GC_B and Th2-associated interactions, whereas Case 2 shows a shift toward increased connectivity involving macrophage-associated populations, including interactions with fibroblasts. These patterns are consistent with remodeling of the immune microenvironment and the emergence of a fibroinflammatory niche. Interactions were inferred from spatial transcriptomic data and should be interpreted as potential cell–cell communication.
