# Supplementary figures and images for: Spatial transcriptomics reveals an SPP1-centered immune–fibrotic axis associated with fibrosis-related tissue remodeling in IgG4-related disease
Source: Front Immunol. 2026 Jul 17;17:1870169. doi: 10.3389/fimmu.2026.1870169 (PMC13424632; doi:10.3389/fimmu.2026.1870169)

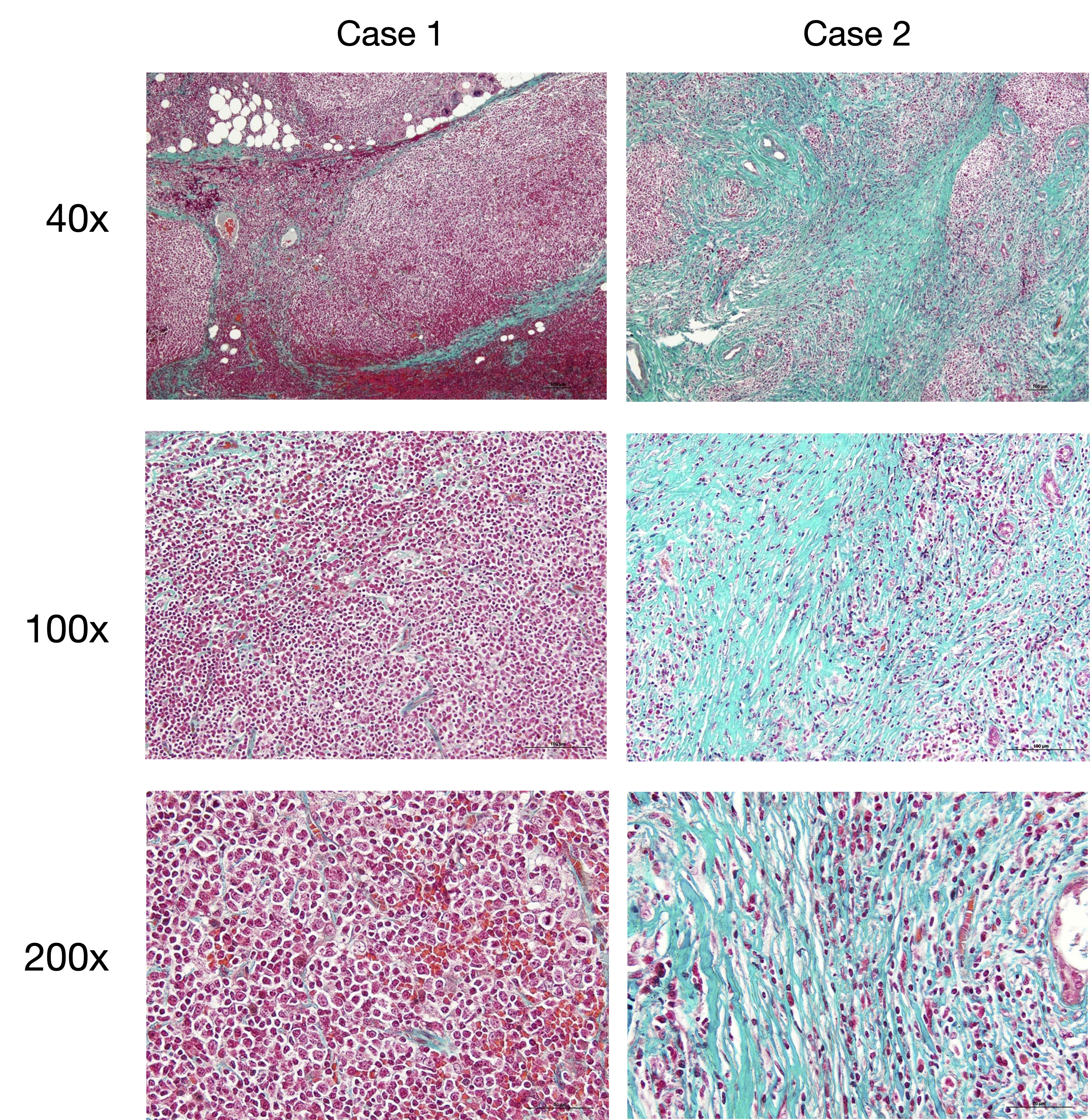

Supplement: Supplementary Figure 1 — Histopathological validation of fibrosis classification by Masson Trichrome staining. Representative Masson Trichrome staining images of the fibrosis-low lesion (Case 1) and fibrosis-high lesion (Case 2). The fibrosis-high lesion showed substantially greater collagen deposition than the fibrosis-low lesion, consistent with the classification based on COL1A1 expression and fibroblast module scores derived from spatial transcriptomic analysis. [file Image1.png]

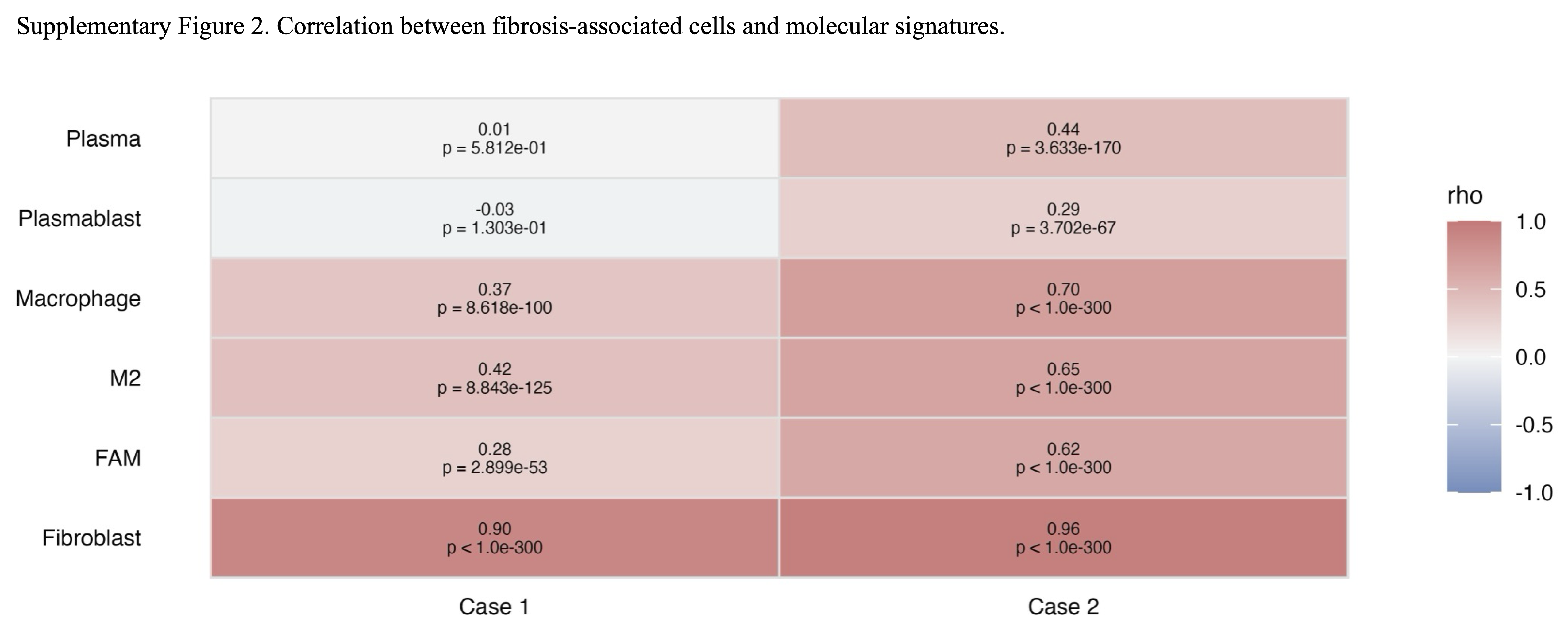

Supplement: Supplementary Figure 2 — Correlation between fibrosis-associated cells and molecular signatures. Heatmap showing Spearman correlation coefficients (rho) between fibrosis-related cellular modules (macrophage, M2, FAM, fibroblast) and plasma cell–related populations in Case 1 and Case 2. Fibroblast and macrophage-associated modules show progressively stronger positive correlations in Case 2, consistent with enhanced coupling between immune and fibrotic programs during disease progression. [file Image2.png]

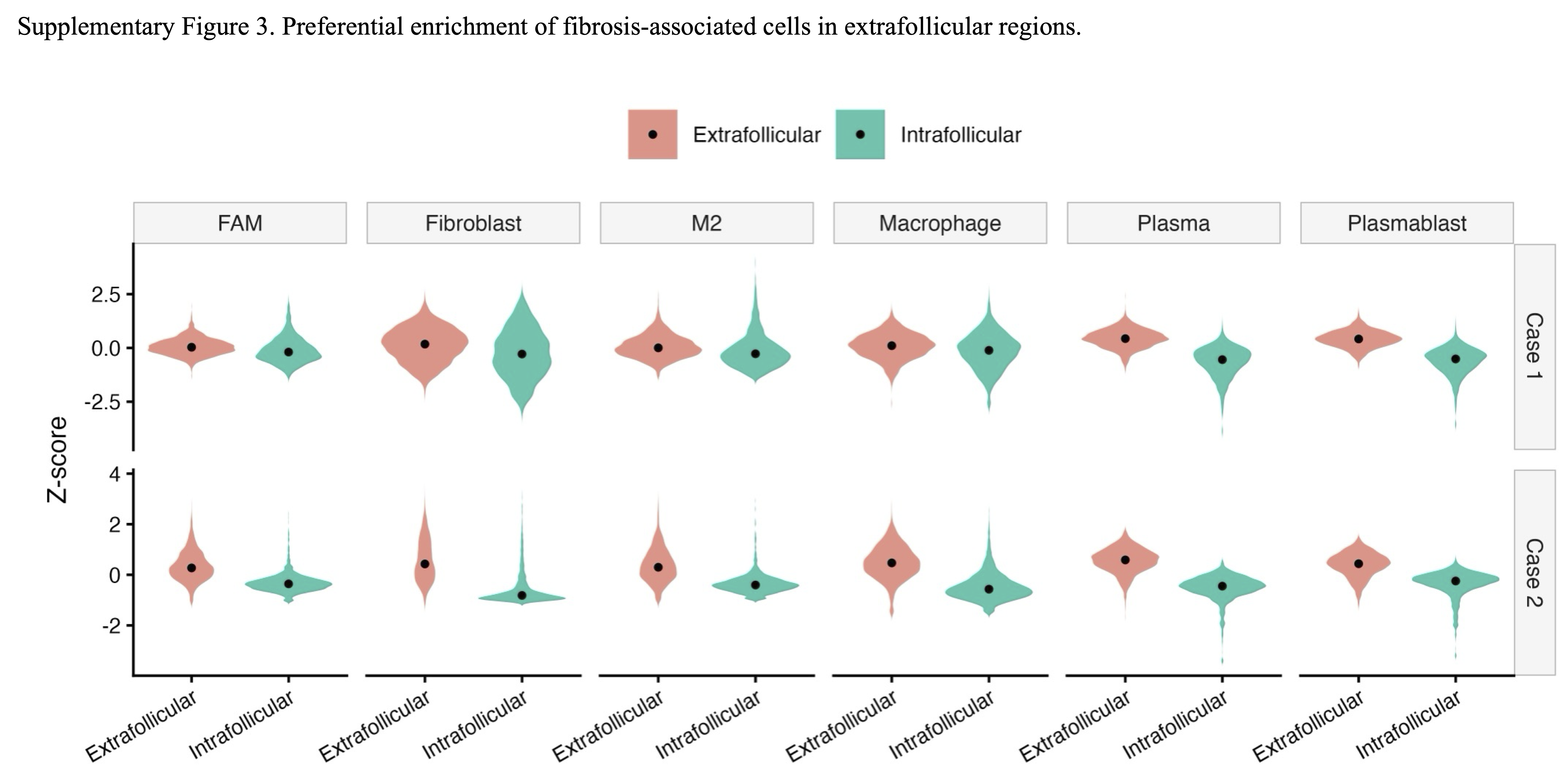

Supplement: Supplementary Figure 3 — Preferential enrichment of fibrosis-associated cells in extrafollicular regions. Violin plots comparing module scores of fibrosis-associated cell populations (FAM, fibroblast, M2, macrophage, plasma cells, and plasmablasts) between intrafollicular and extrafollicular regions in Case 1 and Case 2. Across both cases, but more prominently in Case 2, these cell populations are enriched in extrafollicular regions, supporting the association between fibrosis and extrafollicular immune responses. [file Image3.png]

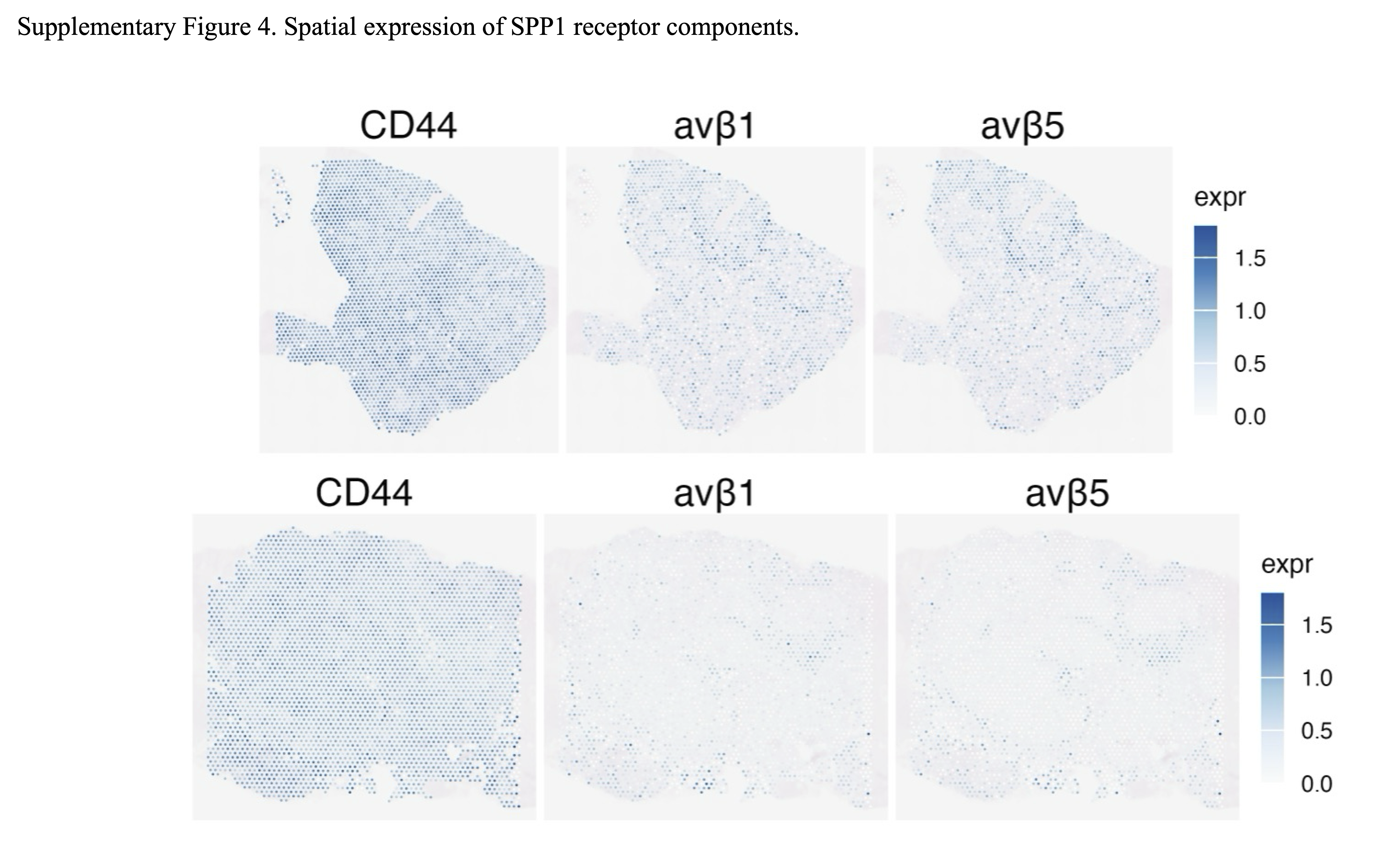

Supplement: Supplementary Figure 4 — Spatial expression of SPP1 receptor components. Spatial expression maps of CD44 and integrin αvβ receptors (αvβ1 and αvβ5) in Case 1 and Case 2. These receptors display overlapping spatial patterns with fibrosis-associated regions, supporting the presence of a functional SPP1 signaling axis within the fibrotic niche. [file Image4.png]

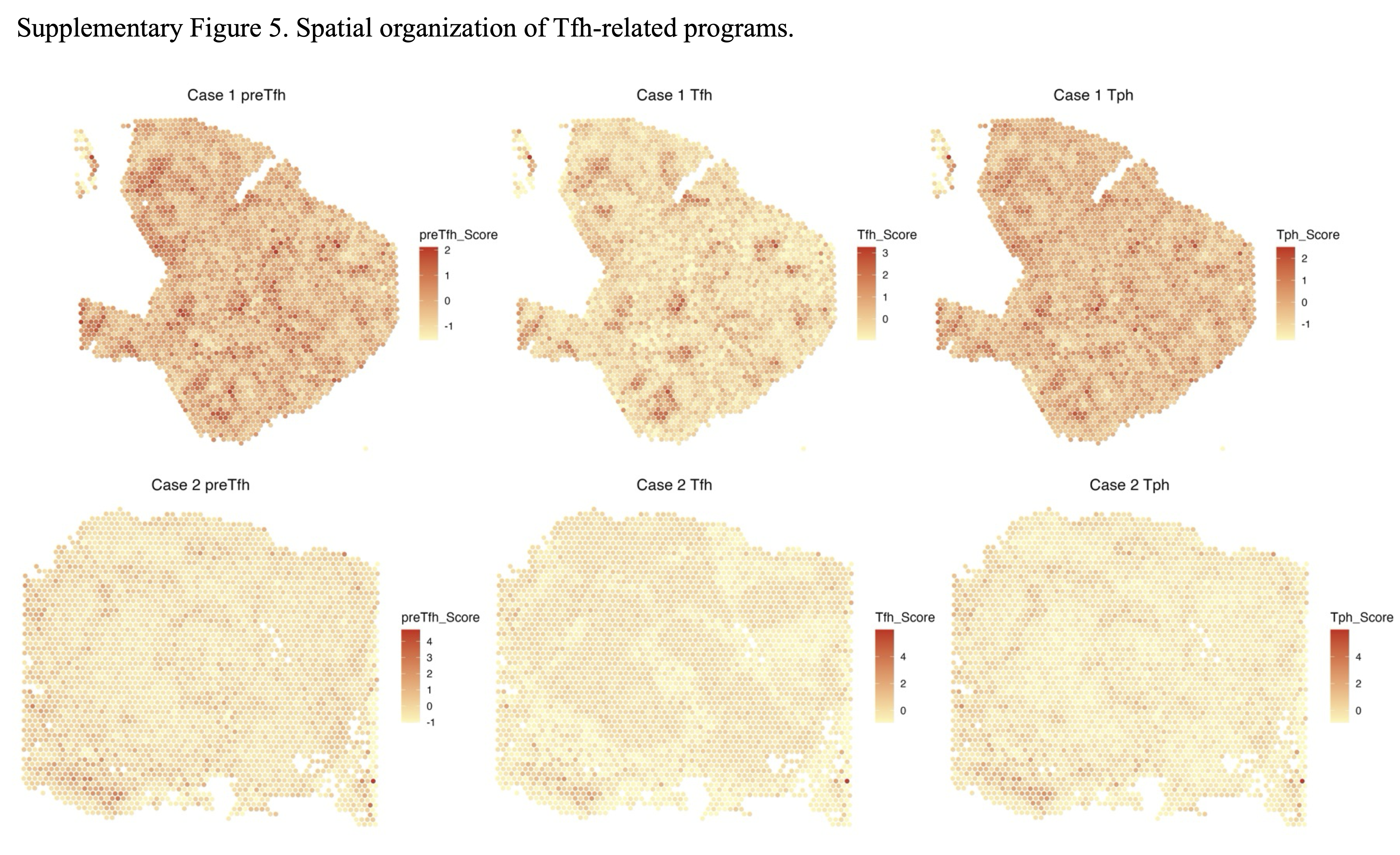

Supplement: Supplementary Figure 5 — Spatial organization of Tfh-related programs. Spatial distribution of preTfh, Tfh, and Tph module scores. In the early case, these programs are broadly distributed, consistent with active immune remodeling, whereas in the advanced case their overall intensity is reduced, suggesting diminished canonical Tfh organization despite strong GC-like features. [file Image5.png]

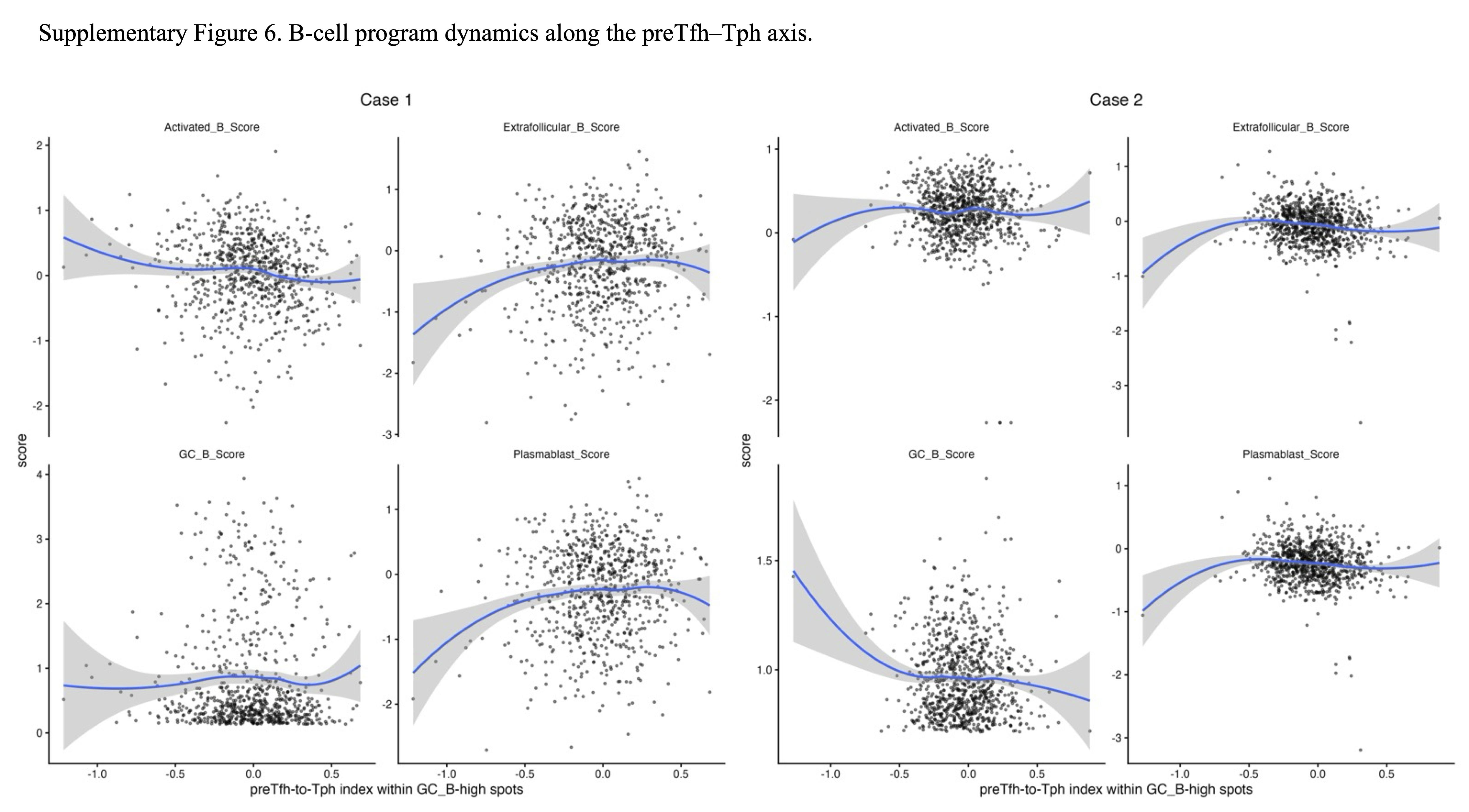

Supplement: Supplementary Figure 6 — B-cell program dynamics along the preTfh–Tph axis. B-cell–related module scores along the preTfh-to-Tph index. In Case 2, increasing preTfh-to-Tph index was associated with reduced GC_B signatures and relative enhancement of extrafollicular B-cell and plasmablast-related programs, supporting a shift in B-cell differentiation states. [file Image6.png]

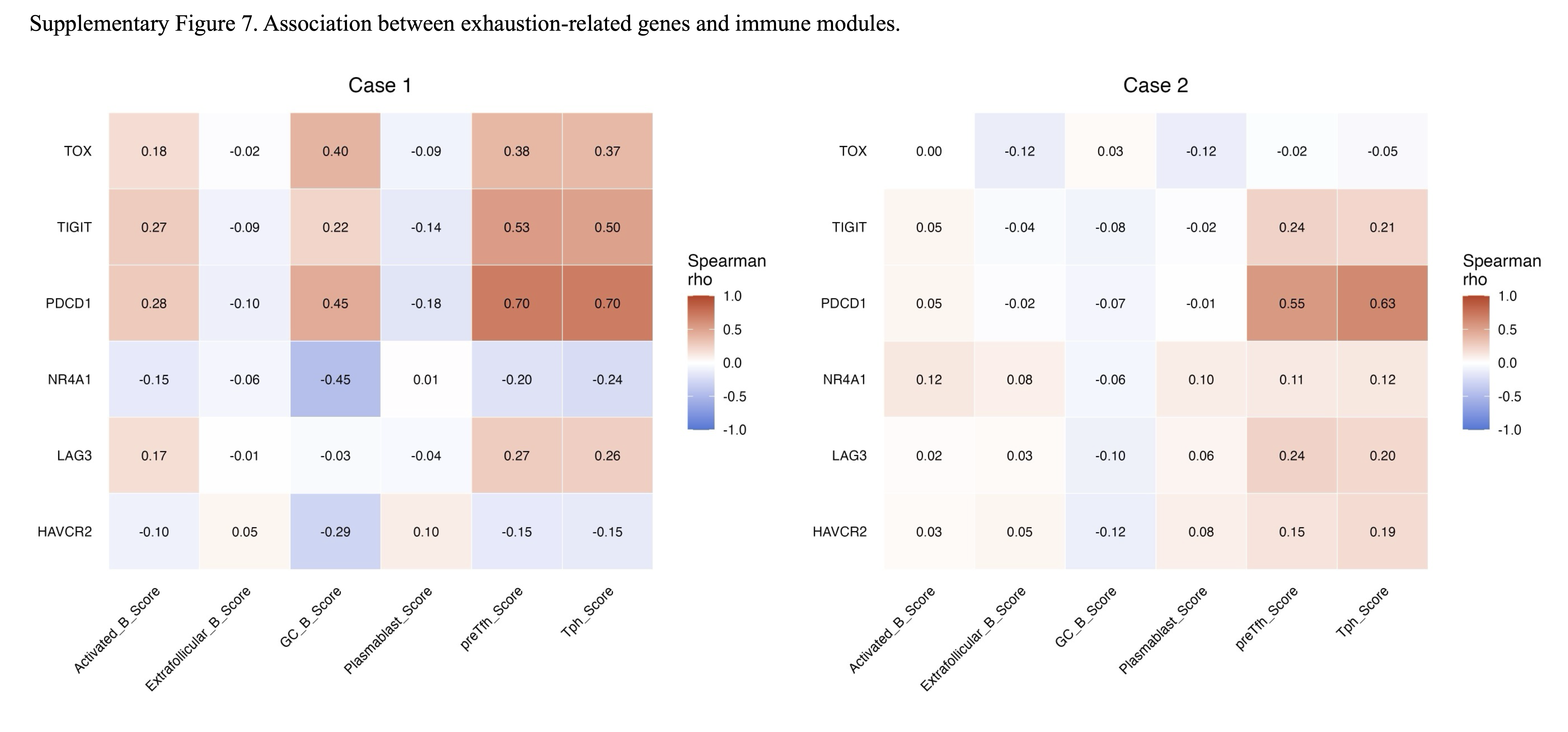

Supplement: Supplementary Figure 7 — Association between exhaustion-related genes and immune modules. Spearman correlation heatmaps between exhaustion-related genes (including TOX, PDCD1, TIGIT, NR4A1) and immune module scores. In the early case, TOX and related exhaustion markers are positively associated with preTfh/Tph programs and B-cell modules, whereas these associations shift toward Tph-related programs in the advanced case, suggesting dynamic reconfiguration of exhaustion-related programs during immune microenvironmental remodeling. [file Image7.png]

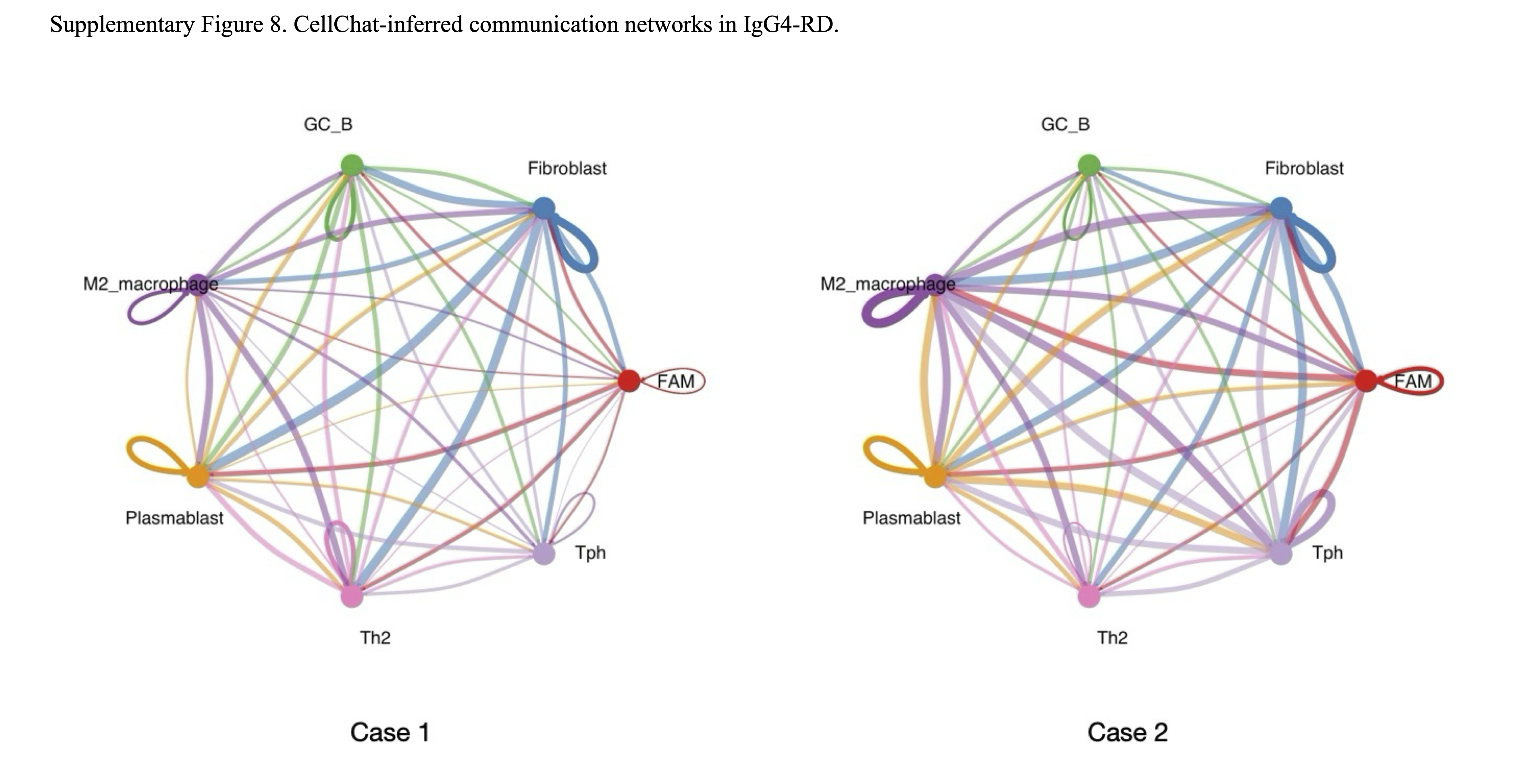

Supplement: Supplementary Figure 8 — CellChat-inferred communication networks in IgG4-RD. (A, B) Inferred interaction networks in Case 1 (A) and Case 2 (B). Nodes represent cell-state groups, and edge thickness indicates inferred communication probability. Case 1 shows a network characterized by prominent GC_B and Th2-associated interactions, whereas Case 2 shows a shift toward increased connectivity involving macrophage-associated populations, including interactions with fibroblasts. These patterns are consistent with remodeling of the immune microenvironment and the emergence of a fibroinflammatory niche. Interactions were inferred from spatial transcriptomic data and should be interpreted as potential cell–cell communication. [file Image8.png]
