# Supplementary material for: Spatial transcriptomics reveals an SPP1-centered immune–fibrotic axis associated with fibrosis-related tissue remodeling in IgG4-related disease
Source: Front Immunol. 2026 Jul 17;17:1870169. doi: 10.3389/fimmu.2026.1870169 (PMC13424632; doi:10.3389/fimmu.2026.1870169)
Supplement: Supplementary file 9 [file DataSheet1.pdf]

Supplementary Table 1. Gene signatures used to define immune and stromal cell modules

Category	Module	Gene symbols
T cell subsets	Th1	TBX21, IFNG, CXCR3, STAT1, IL12RB2
	Th2	GATA3, IL4, IL13
	Th17	RORC, CCR6, IL23R, IL17A
	Treg	FOXP3, IL2RA, CTLA4, IKZF2
	pre-Tfh	CXCR5, ICOS, PDCD1, IL21
	Tfh	CXCR5, BCL6, SH2D1A
	Tph	ICOS, MAF, ZEB2
	CTL	CD8A, LCK, TRBC1
Innate immune cells	NK	NKG7, KLRD1, KLRC1, FCGR3A, GNLY
B cell lineage	B cell	CD79A, CD79B, MS4A1, CD37, CD19
	Germinal center (GC)	BCL6, AICDA, MEF2B, RGS13
	Extrafollicular (ExGC)	IRF4, PRDM1, CXCL12, TNFRSF17
	Plasmablast	PRDM1, XBP1, IRF4, MZB1
	Plasma cell	SDC1, MZB1, XBP1, IGHG
Macrophage lineage	Pan-macrophage	CD68, CSF1R, LST1, AIF1
	M1	IL1B, TNF, CXCL9, CXCL10
	M2	CD163, MRC1, MSR1, TGFB1
	Fibrosis-associated macrophage (FAM)	CD163, MRC1, CCL18, TGFB1
Stromal / fibrosis-related	Fibroblast	COL1A1, COL3A1, FN1, POSTN, FAP, BGN, TIMP1
	Fibrosis	COL1A1, COL3A1, FN1, POSTN, TGFB1, ACTA2, TIMP1
Functional states	Exhaustion	TOX, PDCD1, LAG3, TIGIT, HAVCR2

Gene sets used for module score calculation in spatial transcriptomic analysis. Each module represents a cell type or functional state, and module scores were calculated as the average z-score of the listed genes. Modules were retained for downstream analysis only when at least two genes were detected in the dataset.
